# Wild Bitter Melon Extract Regulates LPS-Induced Hepatic Stellate Cell Activation, Inflammation, Endoplasmic Reticulum Stress, and Ferroptosis

**DOI:** 10.1155/2021/6671129

**Published:** 2021-06-22

**Authors:** Chang-Hsun Ho, Jen-Hsuan Huang, Maw-Sheng Sun, I-Shiang Tzeng, Yi-Chiung Hsu, Chan-Yen Kuo

**Affiliations:** ^1^Department of Anesthesiology, Show Chwan Memorial Hospital, Changhua, Taiwan; ^2^Department of Nursing, Meiho University, Pingtung, Taiwan; ^3^Department of Research, Taipei Tzu Chi Hospital, Buddhist Tzu Chi Medical Foundation, New Taipei City, Taiwan; ^4^Department of Biomedical Sciences and Engineering, National Central University, Taoyuan, Taiwan

## Abstract

The activation of hepatic stellate cells (HSCs) is a key component of liver fibrosis. Two antifibrosis pathways have been identified, the reversion to quiescent-type HSCs and the clearance of HSCs through apoptosis. Lipopolysaccharide- (LPS-) induced HSCs activation and proliferation have been associated with the development of liver fibrosis. We determined the pharmacological effects of wild bitter melon (WM) on HSC activation following LPS treatment and investigated whether WM treatment affected cell death pathways under LPS-treated conditions, including ferroptosis. WM treatment caused cell death, both with and without LPS treatment. WM treatment caused reactive oxygen species (ROS) accumulation without LPS treatment and reversed the decrease in lipid ROS production in HSCs after LPS treatment. We examined the effects of WM treatment on fibrosis, endoplasmic reticulum (ER) stress, inflammation, and ferroptosis in LPS-activated HSCs. The western blotting analysis revealed that the WM treatment of LPS-activated HSCs induced the downregulation of the connective tissue growth factor (CTGF), *α*-smooth muscle actin (*α*-SMA), integrin-*β*1, phospho-JNK (p-JNK), glutathione peroxidase 4 (GPX4), and cystine/glutamate transporter (SLC7A11) and the upregulation of CCAAT enhancer-binding protein homologous protein (CHOP). These results support WM as an antifibrotic agent that may represent a potential therapeutic solution for the management of liver fibrosis.

## 1. Introduction

Chronic liver fibrosis is a health problem, characterized by severe morbidity and significant mortality [1–3]. The underlying physiology of chronic liver fibrosis has been associated with the rapid activation and transdifferentiation of quiescent HSCs fibrogenic myofibroblast-like cells following liver injury or the development of liver fibrosis [4, 5], resulting in cell proliferation, migration, extracellular matrix (ECM) accumulation [6], contraction, chemotaxis, and inflammatory signaling [7]. ECM accumulation has been associated with the increased expression of *α-*smooth muscle actin (*α*-SMA), type I and III collagens, and tissue inhibitor of metalloproteinase-1 (TIMP-1), following the development of liver fibrosis [5, 8–10]. The contraction of HSCs has been proposed to mediate fibrosis by regulating sinusoidal blood flow and ECM remodeling [11]. Recent studies have shown that HSCs are activated by external signals, contributing to liver inflammation or liver injury by producing inflammatory cytokines and directing T lymphocytes into the parenchyma [12]. Multiple cellular and molecular signaling pathways are involved in the regulation of HSC activation: (1) the release of mitogenic (transforming growth factor-alpha (TGF-*α* [13]), platelet-derived growth factor (PDGF [14, 15]), interleukin-1 (IL-1 [16]), tumor necrosis factor-alpha (TNF-*α* [17]), and insulin-like growth factor (IGF-1)) and fibrogenic (transforming growth factor-beta (TGF-*β* [18]) and interleukin-6 (IL-6)) cytokines; (2) receptor activation, including Toll-like receptors (TLRs [19]), collagen receptors [20], liver *X* receptor [21], the nuclear receptor Rev-erb*α* [22], the orphan nuclear receptor NR4A1 [23], vitamin D receptor [24], and G protein-coupled receptors, such as succinate dehydrogenase-G protein-coupled receptor 91 (GPR91) [25, 26]; (3) autophagy, endoplasmic reticulum (ER) stress, and oxidative stress [27–31]; and (4) inflammatory cells, including macrophages, natural killer cells, hepatocytes, and B cells [5, 16, 17, 32–34].

The resolution of hepatic fibrosis requires the clearance of activated HSCs, via apoptosis, or the reversion of HSCs an inactivated phenotype [7–9]. Therefore, HSCs represent an attractive target for antifibrotic therapy [35, 36]. The differentiation of HSCs into proliferative, fibrogenic myofibroblasts is well-known to play a critical role during hepatic fibrosis, as demonstrated by both experimental and clinical human liver injuries [7]. Existing antifibrotic strategies include decreasing the number of activated HSCs via the inhibition of proliferation, the induction of apoptosis, and the inhibition of excessive ECM deposition [37]. Thus, the suppression of HSC growth and/or the induction of HSC apoptosis by natural products are considered to be effective options for the amelioration of liver fibrosis.

Wild bitter melon (WM; *Momordica charantia* L. var. Abbreviata Seringe) is a wild variety of bitter melon (*Momordica charantia*) [38, 39]. The ethyl acetate (EA) fraction from WM has been reported to exhibit strong antioxidant activity, via the scavenging activity of 1, 1-diphenyl-2-picryl-hydrazyl (DPPH), which can reduce H_2_O_2_-induced DNA damage. Moreover, the EA fraction effectively inhibited *α*-amylase activity and suppressed the production of the proinflammatory mediator nitric oxide (NO) in LPS-stimulated murine macrophage RAW 264.7 cells [40]. Similar results indicated that the EA extract of WM suppressed *Propionibacterium acnes*-induced inflammation in THP-1 cells [41]. WM has been demonstrated to have anticancer activities in various cancers types, in vitro, including breast cancer [42–44], colon cancer [45, 46], pancreatic cancer [47], liver cancer [48, 49], prostate cancer [50, 51], cervical cancer [52], and others [53]. However, the full impact of WM on human health has not been thoroughly demonstrated, and systematic clinical studies remain necessary to establish the efficacy and safety of WM extract use in patients. Both in vitro and in vivo studies have demonstrated that bitter melon may elicit toxic or adverse effects under various conditions [53].

This study aimed to investigate whether WM extracts attenuated HSC T6 cell activation induced by LPS treatment. Our data indicated that WM treatments increased ROS and lipid ROS accumulation, induced ER stress, and triggered ferroptosis in LPS-treated HSC T6 cells. We proposed that WM treatment attenuated the LPS-induced HSC activation via ER stress and ferroptosis.

## 2. Materials and Methods

### 2.1. Reagents

WM extract (WM) was purchased from License Biotec, Co., Ltd. (Taipei, Taiwan). The total phenolic extract (TPE) was obtained as described by Huang et al. [54].

### 2.2. Antibodies

The following antibodies were used for immunofluorescence staining and Western blotting: rabbit polyclonal antibodies against CHOP (#A0854, 1 : 1000 dilution, ABclonal, MA, USA), p-JNK (#AP0808, 1 : 1000 dilution, ABclonal, MA, USA), JNK (#A4867, 1 : 1000 dilution, ABclonal, MA, USA), CTGF (#A11456, 1 : 1000 dilution, ABclonal, MA, USA), *α*-SMA (#A1011, 1 : 1000 dilution, ABclonal, MA, USA), integrin-*β*1 (#A11060, 1 : 1000 dilution, ABclonal, MA, USA), GPX4 (#A1933, 1 : 1000 dilution, ABclonal, MA, USA), SLC7A11 (#A13685, 1 : 1000 dilution, ABclonal, MA, USA), and *β*-actin (#AC026, 1 : 5000 dilution, ABclonal, MA, USA).

### 2.3. Cell Culture

HSC-T6, a rat HSC cell line, was purchased from Millipore (MA, USA). HSC-T6 cells were cultured at 37°C in Dulbecco's minimum essential medium (DMEM; Gibco, NY, USA), supplemented with 10% fetal bovine serum (FBS) and antibiotics (100 U/ml penicillin, 100 *µ*g/ml streptomycin, and 2.5 *µ*g/ml amphotericin B), in a humidified atmosphere containing 5% CO_2_. The culture medium was replaced every other day. Once the cells reached 70–80% confluency, they were trypsinized and seeded into 6-well or 24-well plastic dishes for further experiments.

### 2.4. Analysis of Cell Viability

Cell viability was measured using WST-1 assay. Cells were seeded at a density of 5 × 10^4^ cells/mL in 24-well plates and cultured in phenol red-free DMEM, containing 0.5% heat-inactivated FBS, for 24 h. Cells were then incubated with 20 *µ*g/ml of WM or 10 *µ*g/ml of LPS, as indicated, for 24 h. WST-1 reagent was then added to the medium and incubated at 37°C for 2 h. The absorbance was measured at 450 nm using a microplate reader (Thermo Labsystems, Waltham, MA, USA).

### 2.5. Western Blotting

Sodium dodecyl sulfate-polyacrylamide gel electrophoresis (SDS-PAGE) was performed using 10% acrylamide gels, with 20 *µ*g of protein loaded into each lane. After electrophoresis, the proteins were transferred from the gel to a polyvinylidene fluoride (PVDF) membrane, at 350 mA for 2 hours, and the membrane was then blocked with 5% nonfat milk for 1 hour. The membranes were incubated with primary antibodies, diluted 1 : 1,000 in 5% nonfat milk, overnight at 4°C. Membranes were washed in TBST buffer (20 mM Tris-HCl, pH 7.4, 137 mM NaCl, and 0.1% Tween-20) 3 times, for 10 minutes each time, incubated with secondary antibodies conjugated to horseradish peroxidase (HRP), at 1 : 10,000 dilution, for 1 hour at room temperature, washed again, and stained with a Western HRP substrate. Protein bands were visualized on X-ray film using an enhanced chemiluminescence system (Kodak).

### 2.6. Lipid ROS Detection

Cells were incubated with 2 *µ*M C11-BODIPY 581/591 (Thermo Fisher Scientific), in culture medium for 1 h and then washed with phosphate-buffered saline. After trypsinization, cells were collected and used for flow cytometry (BD Biosciences, San Jose, CA, USA), using an excitation wavelength of 488 nm and an emission wavelength of 517–527 nm.

### 2.7. Statistical Analysis

Continuous data were expressed as the mean ± standard error of the mean. Statistical differences among means from different groups were determined by one-way or a two-way analysis of variance, followed by a Bonferroni post hoc test for continuous variables. *P* values <0.05 were considered significant differences.

## 3. Results

### 3.1. WM Treatment Caused ROS Accumulation and Cell Death

**Figure 1:**
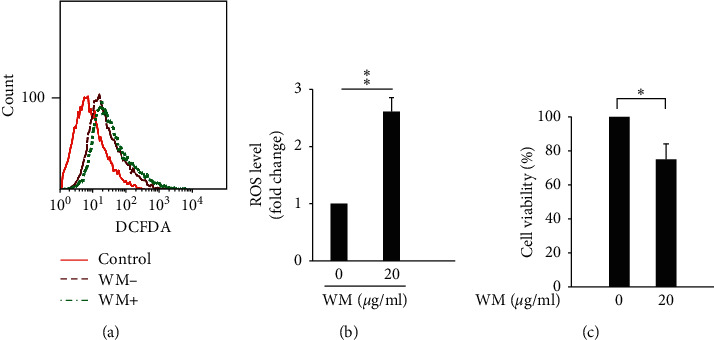
WM treatment induced ROS production and decreased cell viability. (a) Cells treated with (WM+) or without (WM−) 20 *µ*g/ml WM for 24 h. The levels of intracellular ROS were determined using DCF-DA, and fluorescence was detected using FACS Calibur analysis. Control samples refer to cells without DCF-DA. (b) ROS levels are expressed as the mean fluorescence intensity. (c) Cells treated with either the vehicle (0 *µ*g/ml WM) or WM (20 *µ*g/ml) for 24 h. After the incubation period, cell viability was determined using WST-1 assay. All data are presented as the mean ± SD. ^*∗*^*p* < 0.05, ^*∗∗*^*p* < 0.01, *n* = 3.

The acceleration ROS accumulation has been shown to disrupt redox homeostasis and cause severe damage in cancer cells, resulting in cancer cell death via the activation of apoptosis, autophagic cell death, and necroptosis [55]. The induction of apoptosis on HSCs via the stimulation of ROS accumulation represents a potential strategy for addressing liver fibrosis [56]. Our results showed that WM treatment induced ROS overproduction in HSCs relative to untreated cells (Figures 1(a) and 1(b)). Decreased HSC viability was detected after treatment with 20 *µ*g/ml WM for 24 h compared with untreated cells (Figure 1(c)). These results indicated that WM treatment induced ROS accumulation and cell death.

### 3.2. WM Treatment Resulted in Lipid ROS Accumulation and Cell Death in LPS-Activated HSCs

**Figure 2:**
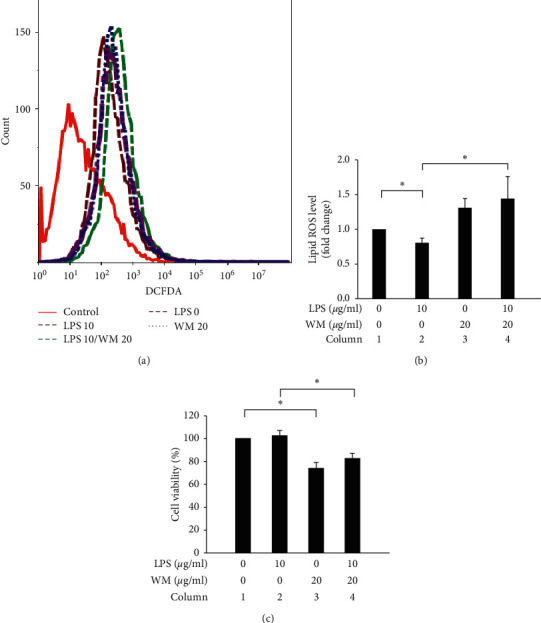
WM treatment reversed the decrease in lipid ROS production and increased cell viability in LPS-activated HSCs. (a) Changes in cellular lipid ROS levels, associated with the indicated conditions in HSC-T6 cells. (b) ROS levels are expressed as mean fluorescence intensity. (c) Cells cultured using the indicated conditions, for 24 h. After the incubation period, cell viability was determined using WST-1 assay. Controls refer cells without 2 *µ*M C11-BODIPY 581/591. LPS 0 indicates cells without LPS treatment. LPS 10 indicates cells treated with 10 *µ*g/ml LPS. WM 20 indicates cells treated with 20 *µ*g/ml WM. All data are presented as the mean ± SD. ^*∗*^*p* < 0.05, *n* = 3.

LPS is a well-known activator of HSCs and LPS treatment results in the activation of a proinflammatory, myofibroblast phenotype [57]. ROS-induced lipid peroxidation and lipid ROS accumulation has been reported to play critical roles in cell death pathways, including apoptosis, autophagy, and ferroptosis [58]. As shown in Figure 2, the results indicated that lipid ROS accumulation decreased in LPS-activated HSCs compared with untreated HSCs (Figures 2(a) and 2(b), column 1 vs. column 2 but increased after the WM treatment of LPS-activated HSCs (WM treatment in LPS-activated HSCs, Figures 2(a) and 2(b), column 2 vs. column 4). In contrast, cell viability significantly decreased after WM treatment in quiescent HSCs compared with untreated cells (Figure 2(c), column 1 vs. column 3). Interestingly, WM treatment caused cell death in LPS-activated HSCs (Figure 2(c), column 2 vs. column 4). Therefore, we proposed that WM treatment resulted in lipid ROS accumulation and cell death in LPS-activated HSCs.

### 3.3. WM Treatment Enhanced ER Stress, Attenuated Inflammation, and Triggered Ferroptosis in LPS-Activated HSCs

**Figure 3:**
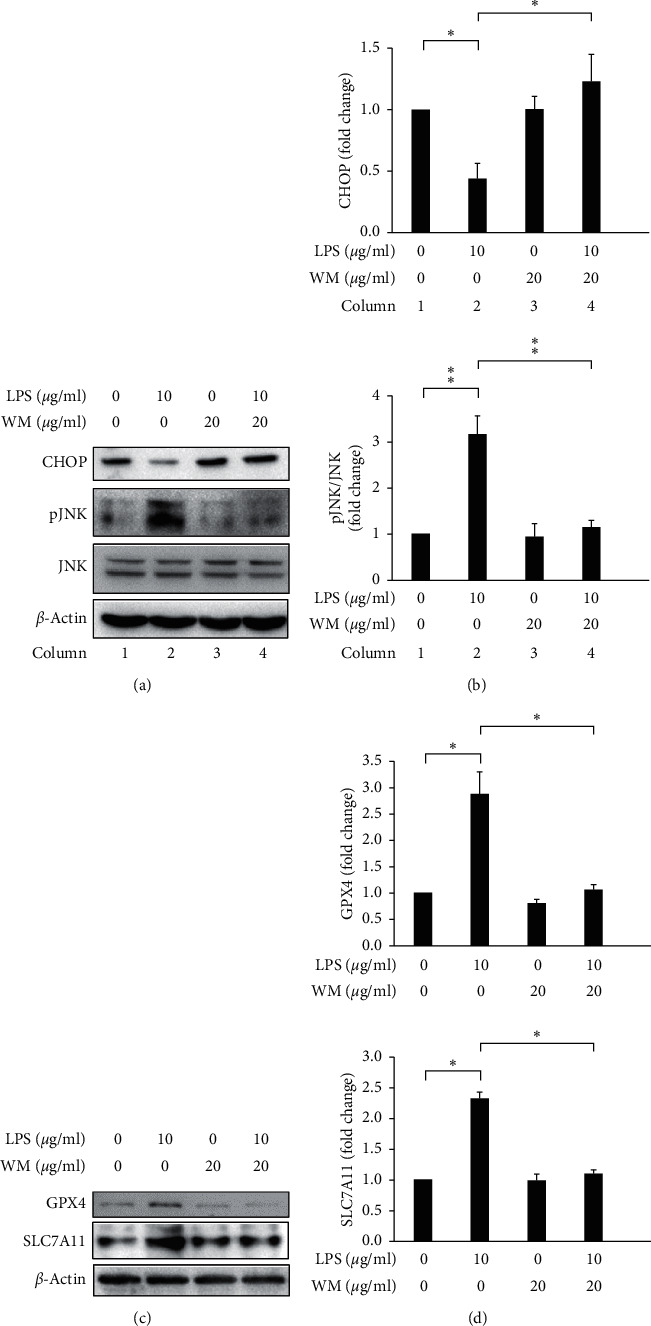
WM treatment induced ER stress, alleviated inflammation, and triggered ferroptosis in LPS-activated HSCs. (a) Changes in the expression levels of CHOP and p-JNK. *β*-Actin was used as an internal control. (b) Quantitative evaluation of the levels of specific proteins, assessed by ImageJ. All data are presented as the mean ± SD. *n* = 3, ^*∗*^*p* < 0.05. (c) Changes in the expression levels of GPX4 and SLC7A11. *β*-Actin was used as an internal control. (d) Quantitative evaluation of the levels of specific proteins, assessed by ImageJ. All data are presented as the mean ± SD. *n* = 3, ^*∗*^*p* < 0.05.

CHOP plays a critical role in ER stress-induced apoptosis [59]. Oyadomari and Mori demonstrated that when severe ER stress conditions persist, apoptotic signaling pathways become activated, resulting in the induction of CHOP [60]. Our results showed that CHOP expression levels decreased in LPS-activated HSCs (Figures 3(a) and 3(b), column 1 vs. column 2) but increased following the WM treatment of LPS-activated HSCs (WM treatment in LPS-activated HSCs, Figures 3(a) and 3(b), column 2 vs. column 4). Additionally, JNK is a well-known regulator of the inflammatory response [61]. As shown in Figures 3(a) and 3(b), the expression levels of p-JNK increased in LPS-activated HSCs, compared with quiescent HSCs (Figures 3(a) and 3(b), column 1 vs. column 2) and decreased after the WM treatment of LPS-activated HSCs (WM treatment in LPS-activated HSCs, Figures 3(a) and 3(b), column 2 vs. column 4).

Ferroptosis is a newly identified cell death pathway, which occurs in an iron-dependent manner and is characterized by iron accumulation and lipid peroxidation during the cell death process [62]. SLC7A11 is a key regulator of the antioxidant system Xc^−^, which mediates the exchange of cysteine and glutamate, and is widely distributed in the phospholipid bilayer [63]. GPX4 (glutathione peroxidase 4) activity decreases with increasing system Xc^−^ activity, resulting in decreased antioxidant capacity, lipid ROS accumulation, and ultimately, oxidative damage and ferroptosis [62]. Friedmann Angeli et al. reported that knockout of GPX4 caused cell death via ferroptosis, both in vitro and in vivo [64]. In the present study, the results showed that the expression levels of GPX4 and SLC7A11 increased in LPS-activated HSCs, compared with untreated HSCs (Figures 3(c) and 3(d), column 1 vs. column 2), but decreased after the WM treatment of LPS-activated HSCs (WM treatment in LPS-activated HSCs, Figures 3(c) and 3(d), column 2 vs. column 4). Altogether, these results indicated that WM treatment sensitized LPS-activated HSCs ER stress, attenuated inflammation, and triggered ferroptosis.

### 3.4. WM Treatment Has Antifibrotic Effects on LPS-Activated HSCs

**Figure 4:**
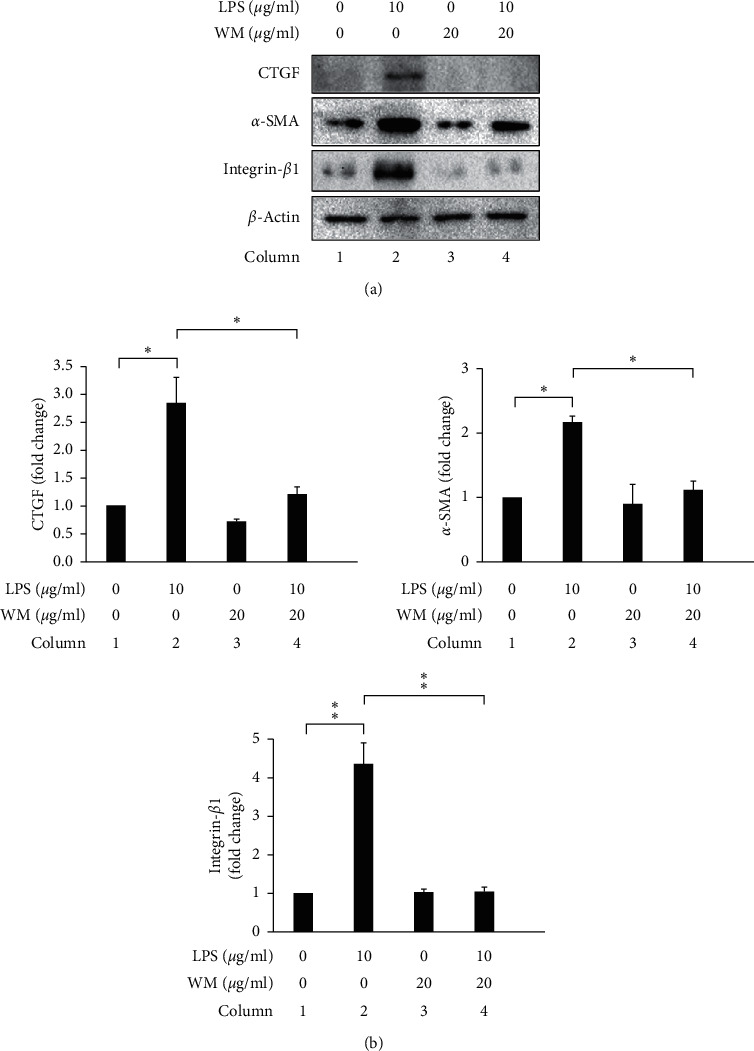
WM treatment attenuated fibrosis in LPS-activated HSCs. (a) Changes in the expression levels of CTGF, *α*-SMA, and integrin-*β*1. *β*-Actin was used as an internal control. (b) Quantitative evaluation of the levels of specific proteins, assessed by ImageJ. All data are presented as the mean ± SD. *n* = 3, ^*∗*^*p* < 0.05, ^*∗∗*^*p* < 0.01.

Activated HSCs are well-known as potential therapeutic targets in liver fibrosis [65]. We investigated whether WM treatments have any antifibrotic effects in LPS-activated HSCs. As shown in Figures 4(a) and 4(b), the expression levels of CTGF, *α*-SMA, and integrin-*β*1 increased in LPS-activated HSCs, compared with untreated HSCs (Figures 4(a) and 4(b), column 1 vs. column 2), but decreased after WM treatment (WM treatment in LPS-activated HSCs, Figures 4(a) and 4(b), column 2 vs. column 4). Therefore, we suggested that WM treatment has great potential for use to treat and prevent liver fibrosis through effects on activated HSCs.

## 4. Discussion

Activated HSCs play major roles in the pathogenesis of liver fibrosis [66]. Growing evidence has suggested that the induction of HSC cell death and the inhibition of HSC growth may represent potential strategies for the treatment and/or prevention of liver fibrosis [9, 33, 67–70]. Furthermore, natural fruits may be used as additional therapeutic approaches to inhibit hepatic fibrogenesis. To our knowledge, this study demonstrated, for the first time, that an extract from the natural fruit WM could attenuate LPS-induced HSC activation via the regulation of ER stress and ferroptosis. However, the pharmacological effects of WM treatments in HSCs remain unclear. Our results indicated that WM treatment caused ROS accumulation, lipid ROS accumulation, and cell death in LPS-activated HSCs (Figures 1 and 2). WM treatment also increased ER stress-induced apoptosis and attenuated inflammation and ferroptosis in LPS-activated HSCs (Figure 3). We also detected the effects of WM treatment on the expression of the following proteins: *α*-SMA, a critical marker of HSC activation [71]; CTGF, a maker of liver fibrosis [72, 73]; and integrin-*β*1, a hallmark of hepatic fibrosis [74]. As shown in Figure 4, WM treatment decreased the expression levels of these proteins (Figure 4). Therefore, these data demonstrated that WM treatment may protect against liver fibrosis via HSC inactivation or death.

Astaxanthin was shown to inhibit liver fibrosis via HSC inactivation and the decreased formation of ECM in carbon tetrachloride (CCl_4_) and bile duct ligation mouse models [75]. Similar results were also reported for treatments with curcumin [76], blueberry [77], silymarin [78], 3, 5-diethoxy-3′-hydroxyresveratrol [79], quercetin [80], epigallocatechin-3-gallate [81], coffee [82], and vitamins [83]. Additionally, Kuo et al. suggested that the marine extract from a gorgonian coral *Pinnigorgia* sp. (Pin) could induce apoptosis in HSC-T6 cells via ROS-ERK/JNK-caspase-3 signaling and may exhibit therapeutic potential for the clearance of HSCs [84]. Other studies have reported similar results [85–87]. These studies further strengthen the evidence for the use of bioactive food components and natural products with potential antifibrotic effects in therapeutic approaches designed to slow or reverse the development of liver fibrosis.

Huang et al. reported that the quantitative high-performance liquid chromatography analysis of WM TPE revealed gallic, chlorogenic, caffeic, ferulic, and cinnamic acids, myricetin, quercetin, luteolin, apigenin, and thymol and that WM TPE displayed an anti-inflammatory response against *Propionibacterium acnes*-induced skin inflammation, in vivo [54]. Chen et al. showed that gallic acids attenuated dimethylnitrosamine-induced fibrosis via the regulation of Smad phosphorylation [88]. Chlorogenic acids protect against CCL_4_-induced liver fibrosis through the suppression of oxidative stress in the liver and HSCs [89]. Caffeic and ferulic acids have been shown to prevent liver damage and ameliorate liver fibrosis in CCL_4_-treated rats [90, 91]. Wang et al. demonstrated that trans-cinnamic acid has antiobesity effects in oleic acid- (OA-) induced HepG2 cells and high-fat diet- (HFD-) fed mice [92]; however, the role played by trans-cinnamic acid in HSCs remains unclear. Myricetin modulated the polarization of macrophages via the inhibition of TREM-1-TLR2/4-MyD88 signaling molecules in macrophages and attenuated liver inflammation and fibrosis in a choline-deficient, L-amino acid-defined, high-fat diet-induced nonalcoholic steatohepatitis model [93]. Quercetin caused decreased oxidative stress and inflammation and prevented liver fibrosis via the induction of HSC apoptosis [94]. Li et al. speculated that luteolin exhibits antifibrotic effects in HSCs and liver fibrosis by targeting the AKT/mTOR/p70S6K and TGF*β*/Smad signaling pathways in CCl_4_, dimethylnitrosamine, and bile duct ligation induced animal models of fibrosis and rat HSCs and HSC-T6 cells [95]. A computational approach indicated that apigenin was predicted to have antifibrotic activity [96]. Thymol significantly ameliorated liver injury due to endotoxicity in gastric ulcer rat models [97]; however, the role played by thymol in liver fibrosis remains uncertain.

## 5. Conclusions

**Figure 5:**
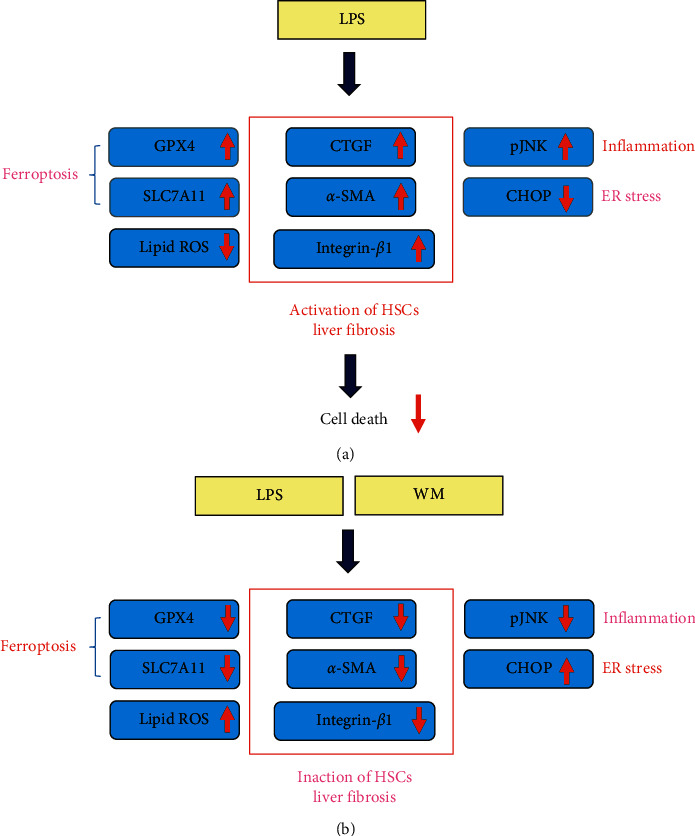
A summary diagram outlines the working mechanisms of WM in LPS-activated HSCs. (a) LPS decreased ER stress and ferroptosis via downregulations of CHOP and GPX4/SLC7A11, respectively. LPS reduced lipid ROS production and cell death, but induced inflammation. On the other hand, LPS promoted liver fibrosis via HSCs activation determined by CTGF, *α*-SMA, and integrin-*β*1 upregulation. (b) WM induced ER stress and ferroptosis via upregulations of CHOP and GPX4/SLC7A11, respectively. WM also caused increases in ROS, lipid ROS production, and cell death, but reduced inflammation. Moreover, WM prevented liver fibrosis via HSCs inactivation determined by CTGF, *α-*SMA, and integrin-*β*1 downregulation.

In summary, the present study demonstrated that the pretreatment of HSCs with WM prevented LPS-induced HSC-T6 cell activation (as demonstrated by CTGF, *α*-SMA, and integrin-*β*1 levels) and inflammation (as indicated by p-JNK levels). WM treatment caused ROS/lipid ROS overproduction, cell death, ER stress activation (as indicated by CHOP expression), and ferroptosis (as indicated by GPX4 and SLC7A11 expression) in LPS-activated HSC-T6 cells (Figure 5). These novel findings deepen our understanding of the mechanistic actions underlying WM treatments. Because WM showed potential antifibrotic effects in activated HSCs, further in vivo studies should be performed to determine the potential effects of WM treatment on various liver fibrosis models.

## Data Availability

The data used to support the findings of this study are included within the article.
